# Chronic Exposure to Low Concentration Lead Chloride-Induced Anxiety and Loss of Aggression and Memory in Zebrafish

**DOI:** 10.3390/ijms21051844

**Published:** 2020-03-07

**Authors:** Ngoc Hieu Bui Thi, Ngoc Anh Nguyen Thi, Gilbert Audira, Petrus Siregar, Sung-Tzu Liang, Jong-Chin Huang, Chung-Der Hsiao

**Affiliations:** 1 Department of Chemistry, Chung Yuan Christian University, Taoyuan 32023, Taiwan; hieubtn90@gmail.com (N.H.B.T.); gilbertaudira@yahoo.com (G.A.); 2 Department of Bioscience Technology, Chung Yuan Christian University, Taoyuan 32023, Taiwan; nguyen021194@gmail.com (N.A.N.T.); siregar.petrus27@gmail.com (P.S.); stliang3@gmail.com (S.-T.L.); 3 Department of Applied Chemistry, National Pingtung University, Pingtung 90003, Taiwan; 4 Center for Biomedical Technology, Chung Yuan Christian University, Taoyuan 32023, Taiwan; 5 Center for Nanotechnology, Chung Yuan Christian University, Taoyuan 32023, Taiwan

**Keywords:** behavior, zebrafish, neurotransmitter, lead, ecotoxicity

## Abstract

Lead and lead-derived compounds have been extensively utilized in industry, and their chronic toxicity towards aquatic animals has not been thoroughly addressed at a behavioral level. In this study, we assessed the risk of exposure to lead at a waterborne environmental concentration in adult zebrafish by behavioral and biochemical analyses. Nine tests, including three-dimension (3D) locomotion, novel tank exploration, mirror biting, predator avoidance, social interaction, shoaling, circadian rhythm locomotor activity, color preference, and a short-term memory test, were performed to assess the behavior of adult zebrafish after the exposure to 50 ppb PbCl_2_ for one month. The brain tissues were dissected and subjected to biochemical assays to measure the relative expression of stress biomarkers and neurotransmitters to elucidate the underlying mechanisms for behavioral alterations. The results of the behavioral tests showed that chronic exposure to lead could elevate the stress and anxiety levels characterized by elevated freezing and reduced exploratory behaviors. The chronic exposure to PbCl_2_ at a low concentration also induced a sharp reduction of aggressiveness and short-term memory. However, no significant change was found in predator avoidance, social interaction, shoaling, or color preference. The biochemical assays showed elevated cortisol and reduced serotonin and melatonin levels in the brain, thus, altering the behavior of the PbCl_2_-exposed zebrafish. In general, this study determined the potential ecotoxicity of long-term lead exposure in adult zebrafish through multiple behavioral assessments. The significant findings were that even at a low concentration, long-term exposure to lead could impair the memory and cause a decrease in the aggressiveness and exploratory activities of zebrafish, which may reduce their survival fitness.

## 1. Introduction

Currently, industries are using lead and lead-derived compounds extensively due to their unique chemical properties and economic values. The application of lead has rapidly grown from five million tons per annum in 1970 to approximately 11 million tons in 2016. However, lead can directly enter aquatic environments from urban sources, including households and waste management and sewage treatment plants. Several studies have reported that lead can cause adverse effects even at a low concentration [1,2]. Dissolved lead concentrations in freshwater and saltwater with chronic effects (EC10/NOEC—usually more than 14 days) on fish range from 18 to 1559 μg/L and 44 to 437 μg/L, respectively [3,4].

More evidence has shown the detrimental impacts of lead on human health, especially at a neurobehavioral level. The neurotoxicity of chronic exposure to low concentrations of lead can impair cognitive performance in childhood through to adulthood [5,6,7,8,9]. Some studies demonstrated the correlation between the exposure to lead at low concentrations and attention-deficit/hyperactivity disorder (ADHD) in children [10]. The development of the central nervous system can be disrupted when children are exposed to low concentrations of lead for a long period of time (blood lead level below 10 mg/dL) [11]. Therefore, lead toxicity has become a serious issue for human health and environmental protection [6]. According to previous studies, the effect of lead on the nervous system includes the disruption of key molecules during neuronal migration and differentiation, interference with synapse formation mediated by a reduction in neuronal sialic acid production and premature differentiation of glial cells, interference with neurotransmitter release, and disruption of the function of GABAergic, dopaminergic, and cholinergic systems [12]. The exposure to lead at high concentrations can cause lead encephalopathy in adults within weeks, with symptoms such as fatigue, headaches, psychomotor agitation, hallucinations, sleep disturbances, delirium, coma, convulsions, attention-deficit, and memory impairment [12,13].

Many studies have also been done in rodent and primate models to assess the effects of early lead exposure on tasks of executive performance, such as spatial working memory, response inhibition, cognitive flexibility, and temporal information processing, which are similar to children with ADHD [10]. An increased distractibility, impulsivity, and an inability to follow sequences of directions in both rats and monkeys provided evidence of lead-induced behavior alterations [14]. Lead-treated monkeys had an impaired ability to organize behavior temporally and were unable to learn from the consequences of previous actions [15]. Recent researches robustly indicated that chronic low-level lead exposure led to the disruption of the pathways related to the exploratory activity in pre-adolescent mice [16]. Six-week-old male mice with 0.2% lead acetate in their drinking water for 12 weeks had deficits in spatial short-term memory and an impairment of adult hippocampal neurogenesis [17].

Although mammalian models have been successfully established to explore the neuronal toxicity of heavy metals, several disadvantages of using rodents as animal models have diverted the attention of the scientific community to other animal models in recent years. Due to the practice of the 3R (Replacement, Reduction, and Refinement) principle regarding animal welfare, the number of rodents used in each experiment must be reduced. However, due to higher individual variation on behavioral performance, a high number of animals should be used to reach statistical significance in behavioral tests. In addition, due to the large space requirement and relatively expensive instrumental setting, the assessment of ecotoxicity by multiple behavioral endpoints using mice has not yet been widely explored. To overcome these limitations, zebrafish have been proposed to serve as an alternative lower vertebrate model for ecotoxicity research.

The use of zebrafish (*Danio rerio*) has several advantages over rodent models, such as easy maintenance, prolific breeding, rapid development, and external development, making them a practical and robust animal model for human diseases and developmental and toxicological studies [11,18,19,20,21]. Several studies have demonstrated the neurotoxic effects of lead exposure at early embryonic stages, thereby causing some changes in the development of the nervous system. Zebrafish embryos treated with lead at 12–72 h post-fertilization (hpf) displayed alterations in neural ventricle formation, hindbrain branchiomotor neurons and neural vasculature, and increased apoptosis [22]. Low-level lead exposure during development resulted in embryotoxicity, behavioral disorders, and learning and memory impairments in zebrafish [6]. Chronic exposure to lead induced learning deficits that persisted for at least three generations, indicating the transgenerational effects of embryonic lead exposure [20]. In addition, a previous study indicated that lead exposure at 10 μmol/L concentration downregulated *nrxn2a* (a synaptic cell-adhesion molecule associated with neurological disorders) expression, increased apoptosis, and altered locomotor behavior in zebrafish embryos and larvae [23].

**Figure 1:**
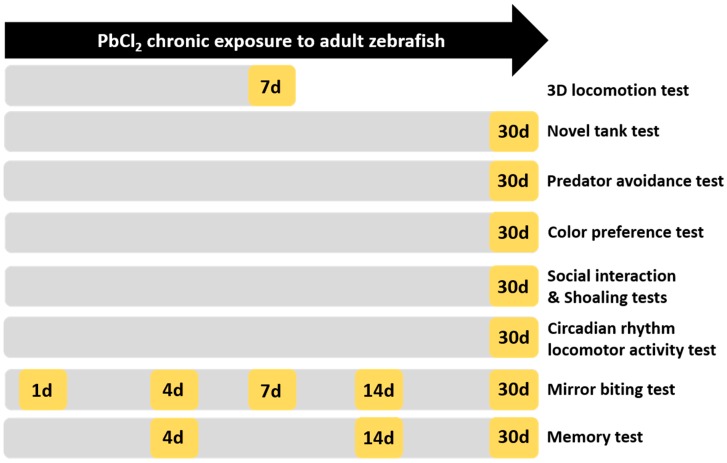
Schematic diagram of the evaluation of the neurobehavioral toxicity of PbCl_2_ in zebrafish. Yellow indicates the time points (day) for conducting behavioral assays. After 30 days, all fish were sacrificed and the biochemical assays were performed.

The potential risks of lead exposure have been extensively studied in the embryonic and larval stages of zebrafish, but the ecotoxicity of lead has not been carefully addressed in adult zebrafish and other aquatic species. In this study, we aim to investigate the toxicity of chronic lead exposure at a low concentration for one month through the assessments of three-dimension (3D) locomotion, novel tank exploration, mirror biting, predator avoidance, conspecific social interaction, shoaling, circadian rhythm locomotor activity, color preference, and short-term memory (the experimental design is illustrated in Figure 1). Biomarkers, including acetylcholine (ACh) and acetylcholinesterase (AChE), gamma-aminobutyric acid (GABA), dopamine, neuroendocrine markers (serotonin and melatonin), and oxidative stress parameters, such as reactive oxygen species (ROS) and superoxide dismutase (SOD), were analyzed to elucidate the underlying mechanism of lead-induced behavioral disorders in adult zebrafish at a cellular and molecular level.

## 2. Results

### 2.1. Lead Exposure Induced Anxiety-Like Behavior in Zebrafish

**Figure 2:**
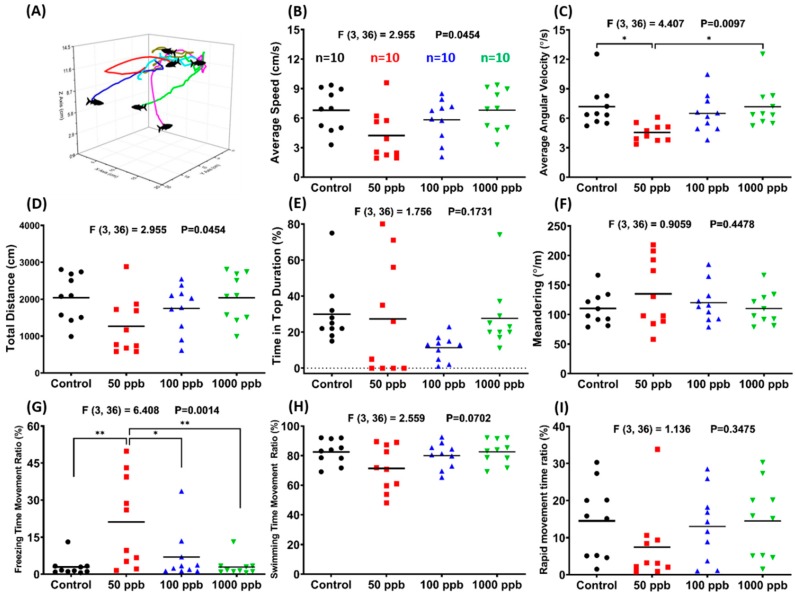
Comparison of three-dimensional (3D) behavior among seven-day PbCl_2_-exposed groups and a control group. (**A**) A schematic diagram showing the typical 3D locomotor paths of several individual fish, using our previously established method. Eight parameters were measured among treatments and a control group: (**B**) average speed, (**C**) average angular velocity, (**D**) total distance, (**E**) time in top duration, (**F**) meandering, (**G**) freezing time movement ratio, (**H**) swimming time movement ratio, and (**I**) rapid movement time ratio. Data are expressed in mean values evaluated by one-way ANOVA (*n* = 10; * *p* ≤ 0.05, and ** *p* ≤ 0.01).

Three-dimensional (3D) locomotion is a sensitive tool for the risk assessment of the alteration of swimming behavior after exposure to chemicals or pollutants [24]. It was found that 50 ppb PbCl_2_-treated zebrafish had the lowest average speed (Figure 2B) and average angular velocity (Figure 2C). The total distance traveled was also decreased in all of the PbCl_2_-treated zebrafish, but no significant difference was found when compared with the control group (Figure 2D). The correlation between rapid movement time ratio and average speed could be identified by a freezing time movement ratio. The 50 ppb PbCl_2_-treated zebrafish had the highest freezing time movement ratio; thus, they also had the lowest rapid movement time ratio. However, there was only a slight decrement in the swimming time movement ratio (Figure 2G–I). Based on these results, lead at its lowest concentration, 50 ppb, can induce anxiety-like behavior in adult zebrafish. Supplementary Video S1 display the behavioral changes for the control and 50 ppb PbCl_2_-treated zebrafish in the 3D locomotion test. 

**Figure 3:**
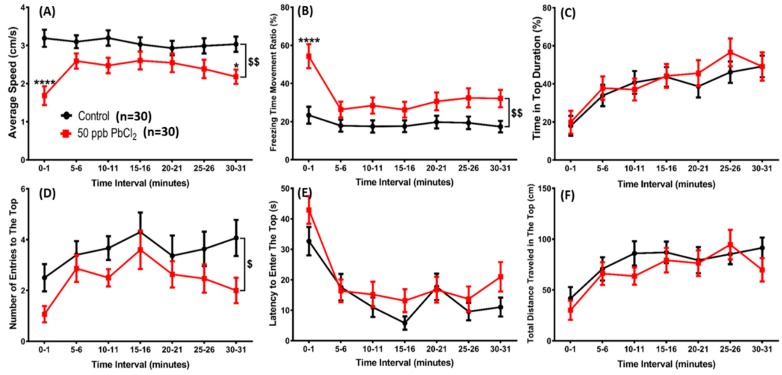
Comparison of behavioral endpoints in the novel tank exploration test among 30-day 50 ppb PbCl_2_-exposed groups and a control group. Six parameters were evaluated: (**A**) average speed, (**B**) freezing time movement ratio, (**C**) time in top duration, (**D**) number of entries to the top, (**E**) latency to enter the top, and (**F**) total distance traveled in the top. Data are expressed as mean ± SEM and analyzed by two-way ANOVA with Sidak’s multiple comparisons test (*n* = 30; */$ *p* ≤ 0.05, $$ *p* ≤ 0.01, and **** *p* ≤ 0.0001).

In addition to the 3D locomotion test, a novel tank exploration test was also performed to determine whether lead exposure induces anxiety through the chronological response assay in a novel environment. We found that the zebrafish exposed to 50 ppb PbCl_2_ displayed reduced locomotor activity (Figure 3A), a higher freezing time movement ratio (Figure 3B), and a lower number of entries to the top compartment (Figure 3D) compared to the control group. Other parameters, including the time (Figure 3C), latency to enter (Figure 3E), and total distance traveled in the top compartment (Figure 3F) of the control and PbCl_2_-exposed fish had no significant difference. The locomotor activity and the number of entries to the top zone in most of the observation time decreased, indicating that exposure to lead at a low concentration does not simply reduce locomotor behavior, but also induces anxiety-like behavior in a new environment. Supplementary Video S2 display the behavioral changes for the control and 50 ppb PbCl_2_-treated zebrafish in the novel tank test.

### 2.2. Lead Exposure Decreased Aggressiveness in Zebrafish

**Figure 4:**
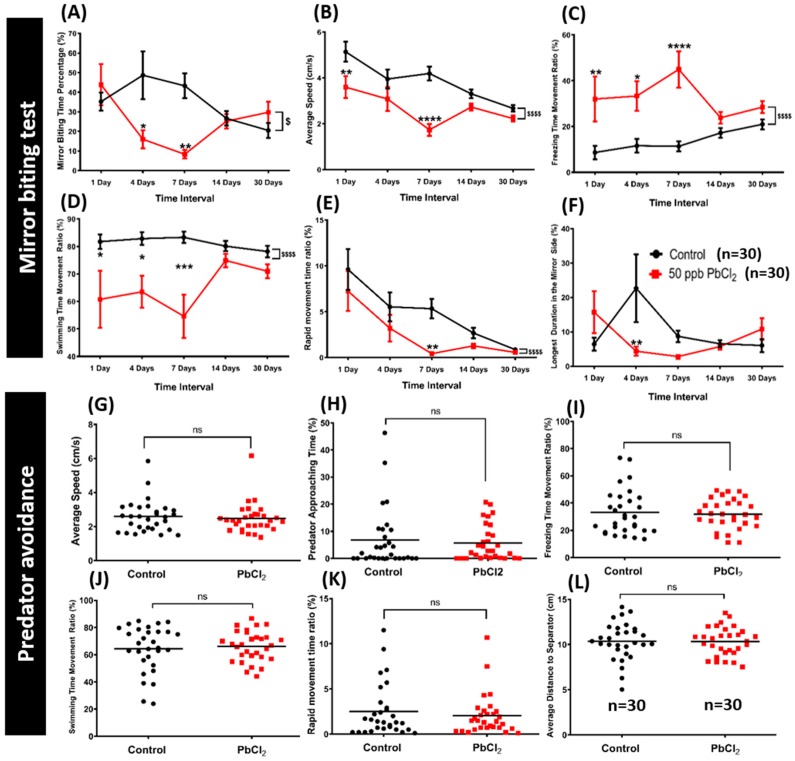
Evaluation of aggressiveness and fear by mirror-biting and predator avoidance tests between the control and 50 ppb PbCl_2_-treated fish. After PbCl_2_ exposure (red line), the mirror biting behavior was chronologically monitored and the data were presented as (**A**) mirror biting time percentage, (**B**) average speed, (**C**) freezing time movement ratio, (**D**) swimming time movement ratio, (**E**) rapid movement time ratio, and (**F**) longest duration in the mirror side. Data are expressed as mean ±SEM values and analyzed by two-way ANOVA with Sidak’s multiple comparisons test (*n* 1 & 7 days = 10, *n* 14 days = 40, *n* 30 days = 30; */$ *p* ≤ 0.05, ** *p* ≤ 0.01, *** *p* ≤ 0.001 and ****/$$$$ *p* ≤ 0.0001). After PbCl_2_ exposure for 30 days, the predator avoidance behavior was monitored and data were presented as (**G**) average speed, (**H**) predator approaching time, (**I**) freezing time movement ratio, (**J**) swimming time movement ratio, (**K**) rapid movement time ratio, and (**L**) average distance to separator. Data are expressed as mean and analyzed by the Mann–Whitney test (*n* = 30).

The mirror biting experiment is a well-established test that is commonly used for studying the social/aggressive behavior of adult zebrafish [25]. Mirror biting behavior and rapid swimming indicates the aggressiveness of zebrafish. In our study, a mirror biting test was performed after lead treatment. We used a chronological approach to measure aggressiveness in the mirror biting test after 1 day, 4 days, 7 days, 14 days, and 30 days of PbCl_2_ exposure. The average speed and mirror biting time percentage of the treated fish were significantly reduced on the seventh day (Figure 4A,B). Furthermore, they displayed anxiety, as indicated, by an increased freezing time movement ratio in most of the time intervals. The most significant change was observed on day 7 of PbCl_2_ incubation, when compared to the control group (Figure 4C). The downward trend of the swimming time movement ratio and rapid movement time ratio demonstrated that the PbCl_2_ treatment had a significant impact on aggressive behavior (Figure 4D,E). In addition, the significantly reduced duration in the mirror side of the treated fish indicated a less aggressive behavior than the control fish (Figure 4F). These results demonstrated that a low concentration of PbCl_2_ decreased aggressiveness in adult zebrafish. Supplementary Video S3 display the behavioral changes for the control and 50 ppb PbCl_2_-treated zebrafish in the mirror biting test. Since PbCl_2_ at 50 ppb induced the most significant anxiety-like response in both the 3D locomotion and mirror biting test, it was considered the optimal concentration; thus, it was used to test other zebrafish behavioral endpoints in the following experiments.

### 2.3. Lead Exposure Did Not Alter Fear Response in Zebrafish

Predator avoidance is an innate response to help the prey to escape from its predator. In this test, we used a convict cichlid (*Amatitlania nigrofasciata*) as a predator fish to induce the fear response of the zebrafish. In avoiding the predator, any unusual movements of the PbCl_2_-treated fish compared to the control fish could be conceived as an impact of the treatment. We found that there were no significant differences in behavioral endpoints of the PbCl_2_-treated fish and the control group (Figure 4G–L). This result demonstrated that chronic exposure of PbCl_2_ at 50 ppb did not alter the predator avoidance response in adult zebrafish. Supplementary Video S4 display the behavioral changes for the control and 50 ppb PbCl_2_-treated zebrafish in the predator avoidance test.

### 2.4. Lead Exposure Did Not Alter Conspecific Interaction and Shoaling Behaviors

**Figure 5:**
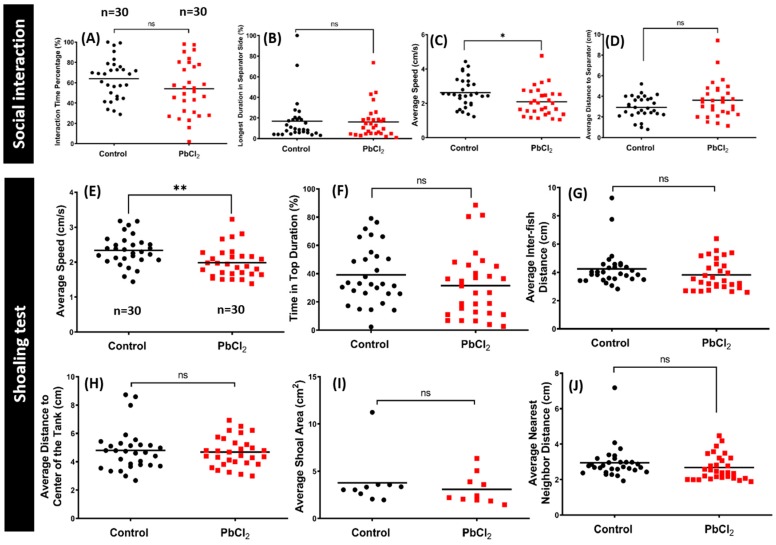
Comparison of the conspecific social interaction and shoaling between control and 50 ppb PbCl_2_-treated fish. After PbCl_2_ exposure, the conspecific social interaction was monitored and the data are presented as (**A**) interaction time percentage, (**B**) longest duration in the separator side, (**C**) average speed, and (**D**) average distance to the separator. The data are expressed as the mean values and analyzed by the Mann–Whitney test (*n* = 30; * *p* ≤ 0.05). For the shoaling test, after PbCl_2_ exposure, the shoaling behavior was monitored at 30 days post-treatment and the data are presented as (**E**) average speed, (**F**) time in the top duration, (**G**) average inter-fish distance, (**H**) average distance to the center of the tank, (**I**) average shoal area, and (**J**) average nearest neighbor distance. Data are expressed as the mean values and analyzed by the Mann–Whitney test (*n* = 30; ** *p* ≤ 0.01).

The social phenotype is an important part of the natural behavior of a zebrafish. We used a conspecific social interaction test to investigate whether chronic lead exposure has any detrimental effects on the social interaction of the zebrafish. Normally, zebrafish display high sociability when placed in a tank with conspecific fish. In our test, the PbCl_2_-treated fish showed no significant alteration in the interaction time percentage (Figure 5A), longest duration in separator side (Figure 5B), or average distance to the separator (Figure 5D) when compared with the control fish. However, the PbCl_2_-exposed fish displayed a significant reduction in locomotor activity compared to the control group, which was consistent with our result in another behavioral test (Figure 5C). Supplementary Video S5 display the behavioral changes for the control and 50 ppb PbCl_2_-treated zebrafish in the social interaction test.

We also performed a shoaling test to assess the anxiogenic effects of PbCl_2_ exposure. Generally, adult zebrafish display a stable shoal cohesion. In this study, we measured six endpoints to evaluate the shoal cohesion of the fish after thirty days of PbCl_2_ exposure. A previous study showed that the fish became more active and began exploration of the top area after a few minutes of acclimation. Furthermore, anxious fish tend to swim together in a tightened shoal compared to non-anxious fish when placed in a novel environment. Therefore, the average speed, time in top duration, average inter-fish distance, and average distance to the center of the tank may reflect the exploration preference or anxiety and stress-like behavior of zebrafish. In addition, other endpoints, such as the average shoal area and average nearest neighbor distance endpoints, allow researchers to study shoal cohesion, since they can demonstrate the anxiogenic effects of an experimental drug. In this study, the PbCl_2_-exposed fish showed no significant difference with the control group in the time in the top duration (Figure 5F), average inter-fish distance (Figure 5G), average distance to the center of the tank (Figure 5H), average shoal area (Figure 5I), and average nearest neighbor distance (Figure 5J). However, the average speed of PbCl_2_-exposed fish was significantly reduced compared to the control fish, which was also consistent with our results from other behavioral tests (Figure 5E). Altogether, exposure to a low concentration of lead did not alter the social interaction and shoaling behavior of zebrafish. However, it is important to note that, based on some of the results, it induced anxiety in zebrafish. Supplementary Video S6 display the behavioral changes for the control and 50 ppb PbCl_2_-treated zebrafish in the shoaling test.

### 2.5. Lead Exposure Altered Zebrafish Circadian Rhythm Locomotor Activity

**Figure 6:**
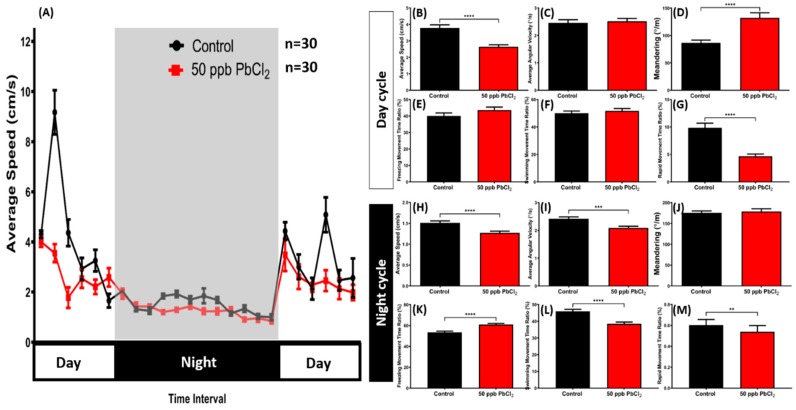
Evaluation of the circadian rhythm locomotor activity for control and chronic PbCl_2_-treated fish (50 ppb, red). (**A**) Circadian locomotor activity patterns of average speed; (**B**) average speed; (**C**) average angular velocity; (**D**) meandering; (**E**) freezing movement time ratio; (**F**) swimming movement time ratio; and (**G**) rapid movement ratio during the light cycle; (**H**) average speed; (**I**) average angular velocity; (**J**) meandering; (**K**) freezing movement time ratio; (**L**) swimming movement time ratio; and (**M**) rapid movement ratio during the dark cycle. The data are expressed as mean ± SEM and analyzed by the Mann–Whitney Test (*n* control fish = 18; *n* PbCl_2_-treated fish = 18; ** *p* < 0.01, *** *p* < 0.001, **** *p* < 0.0001).

In the circadian rhythm locomotor activity test with the light/dark (L/D) 12/12 h setting, the PbCl_2_-exposed fish generally maintained lower locomotor activity than the control fish during both the light and dark cycles (Figure 6A). In the light cycle, this phenomenon was shown by a lower average speed and rapid movement time ratio of the treated fish (Figure 6B,G). Irregular movements were also observed in the treated fish, which was indicated by a high level of meandering during the light cycle (Figure 6D). Meanwhile, there were no significant differences shown in the average angular velocity, freezing movement time ratio, or swimming movement time ratio of the treated group compared with the control group (Figure 6C,E,F). Furthermore, low locomotor activity in the PbCl_2_-exposed fish during the dark cycle was noticed, as indicated by a low average speed, average angular velocity, swimming movement time ratio, and rapid movement time ratio (Figure 6H,I,L,M). Moreover, a significant difference was found in the freezing movement time ratio of the control and the PbCl_2_-treated group (Figure 6K). On the other hand, there was no significant difference in the meandering during the dark cycle (Figure 6J). Overall, based on the results, it can be inferred that a low concentration of PbCl_2_ altered the adult zebrafishes’ locomotor activity in both light and dark cycles.

### 2.6. Lead Exposure Induced Short-Term Memory Loss in Zebrafish

**Figure 7:**
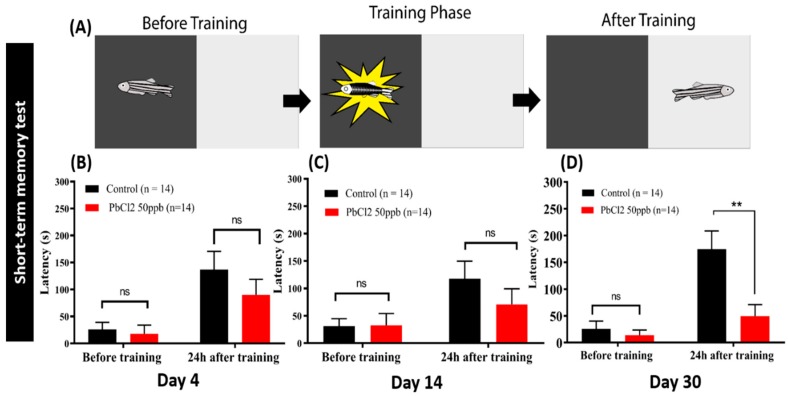
Passive avoidance task to test the short-term memory of fish treated with different exposure durations in 50 ppb of PbCl_2_. (**A**) An illustration that shows the principle of the shuttle box experiment. Twenty-four hours after the training session, we tested the latency for the fish to enter the dark compartment equipped with an electrical shock. Latency of fish treated with PbCl_2_ (50 ppb) after 4 days (**B**), 14 days (**C**), and 30 days (**D**) after training. Data are expressed as mean ± SEM values and analyzed by two-way ANOVA (*n* = 10; ** *p* ≤ 0.01).

The passive avoidance task is a fear-associated test used to assess central nervous system-related disorders in rodent models [26,27]. In this test, a subject learns to escape an aversive stimulus (such as a foot-shock). Therefore, the passive avoidance task is a useful test to evaluate the effect of PbCl_2_ on short-term memory. A shuttle box is one of the apparatuses that provides stimulus condition. It has been applied to determine memory loss induced by metal poisoning. In this study, we used the passive avoidance test in a shuttle box to assess whether exposure with 50 ppb PbCl_2_ could impair short-term memory in adult zebrafish. The results showed that the control fish could maintain short-term memory with a latency time around 150–200 s during the testing phase (Figure 7A–C). Within four days and 14 days, the PbCl_2_-treated fish did not show any significant change in the latency (Figure 7B,C). However, the exposure of PbCl_2_ for one month significantly reduced the latency down to approximately 50 s in the testing session (Figure 7D). It is demonstrated that long-term PbCl_2_ exposure can induce short-term memory loss in zebrafish.

### 2.7. Lead Exposure Did Not Alter Color Preference Sequence

**Figure 8:**
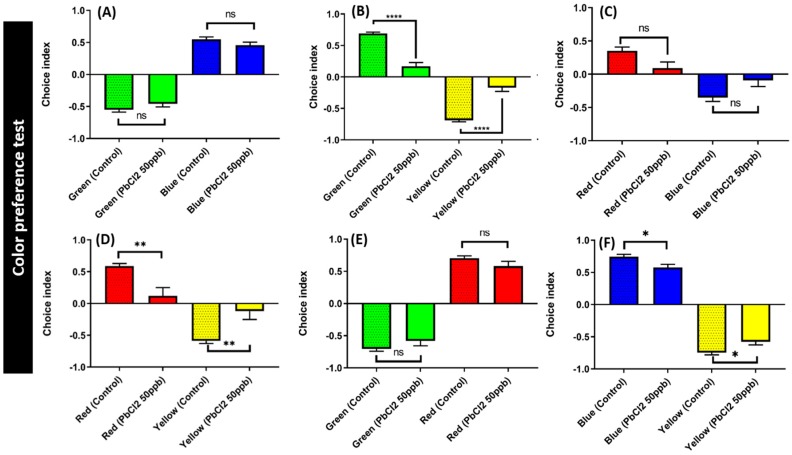
Comparison of the color preference between control and PbCl_2_-exposed fish (50 ppb for 30 days). The combinations of four colors are (**A**) green/blue, (**B**) green/yellow, (**C**) red/blue, (**D**) red/yellow, (**E**) green/red, and (**F**) blue/yellow. Data are expressed as mean ±SEM values and analyzed by two-way ANOVA (*n* = 16; * *p* ≤ 0.05, ** *p* ≤ 0.01, **** *p* ≤ 0.0001).

Zebrafish have multiple cone cell-specific photoreceptors for color discrimination. Chemicals targeting cone cell-specific photoreceptors and brain neurotransmitter expression can induce color preference alteration in zebrafish. We exposed the zebrafish to PbCl_2_ at 50 ppb for 30 days and their color preference was determined according to our previous protocol. Healthy adult zebrafish have a color preference as follows: red > blue > green > yellow (Figure 8). The PbCl_2_-exposed zebrafish did not show any significant changes on color discrimination compared to the control group. However, the color plate combination for green/yellow (Figure 8B), red/yellow (Figure 8D), red/green (Figure 8E), and blue/yellow (Figure 8F) displayed a reduced preference index after PbCl_2_ exposure. When combined with yellow, the red, blue, and green color preference intensities were significantly reduced after long-term PbCl_2_ exposure. In the red/green combination, the PbCl_2_-treated fish also significantly showed a reduction in the preference for red compared to the control group. Meanwhile, the blue-over-green and red-over-blue preferences were not significantly decreased by chronic PbCl_2_ exposure.

### 2.8. Comparison of Biomarkers in the Brain Tissue of the Control and Lead-Treated Zebrafish

**Figure 9:**
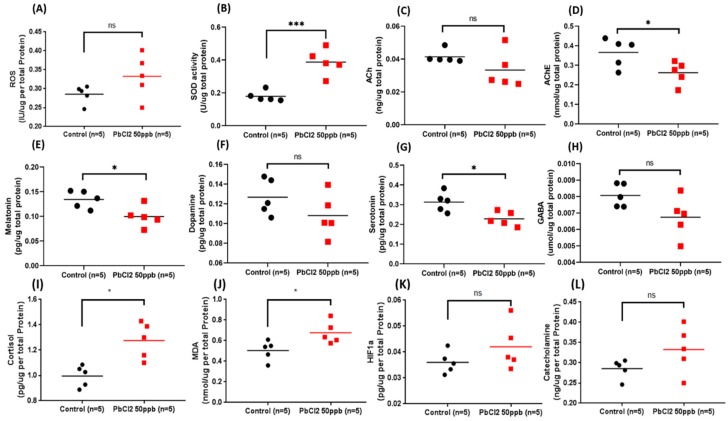
Biochemical parameters of the control and 50 ppb PbCl2-exposed fish. The levels of biomarkers, including (**A**) ROS (reactive oxygen species), (**B**) SOD (superoxide dismutase), (**C**) acetylcholine, (**D**) acetylcholine esterase, (**E**) melatonin, (**F**) dopamine, (**G**) serotonin, (**H**) GABA, (**I**) cortisol, (**J**) MDA (malondialdehyde), (**K**) HIF-1α, and (**L**) catecholamine were measured. Data are expressed as mean values (*n* = 5; * *p* ≤ 0.05, *** *p* ≤ 0.001 tested by *t*-test).

After all the behavioral tests were performed, we sacrificed the fish and their brain tissues were subjected to ELISA for the measurement of neurotransmitter expression. Initially, we evaluated the oxidative stress and lipid peroxidation by measuring the reactive oxygen species (ROS), superoxide dismutase (SOD), and malondialdehyde (MDA) markers. Interestingly, we found that PbCl_2_ exposure at 50 ppb for 30 days could significantly elevate the antioxidant enzyme SOD levels (Figure 9B). In addition, we also noticed a reduction of acetylcholinesterase (AChE) (Figure 9D) in fish after exposure to a low concentration of PbCl_2_ for 30 days. We hypothesized that the reduced AChE and elevated SOD levels may have contributed in the recovery of the behavioral alteration patterns detected in the mirror biting assay by boosting the total antioxidant and anti-dementia activity in the acclimation process. However, there was no significant differences regarding their level of ROS and ACh observed in this experiment (Figure 9A,C).

Melatonin is a hormone that plays an important role in mediating the circadian rhythm in animals, showing high levels in the night cycle and low levels in the day cycle. We determined significantly low levels of melatonin in the PbCl_2_-exposed fish (Figure 9E). This result suggests that the circadian rhythm locomotor activity dysregulation in the PbCl_2_-exposed fish was due to the decrease in melatonin levels. Some of the biomarkers—namely, AChE and serotonin—had also been significantly reduced, whereas the ACh, dopamine, and GABA levels in the PbCl_2_-treated group displayed no significant difference with the control.

## 3. Discussion

In this study, we used multiple behavioral assays, including 3D locomotion, novel tank exploration, mirror biting, predator avoidance, social interaction, shoaling, circadian rhythm locomotor activity, color preference, and short-term memory tests, to evaluate the toxicity of low concentrations of PbCl_2_ in adult zebrafish. The most significant and important finding in our study was the discovery of memory loss in adult zebrafish induced by chronic PbCl_2_ exposure at a concentration of 50 ppb. This finding is consistent with previous publications about the neurotoxicity and memory impairment induced by chronic lead exposure in rodents [28,29,30]. On the other hand, the study done by Chen et al. also showed that the 25 to 50 ppb lead acetate exposure of 8 to 120 hpf zebrafish induced memory deficiency at the adult stage (determined at 150 dpf), which was tested by food reward-based T-maze [6]. In this study, we found that waterborne exposure of PbCl_2_ at 50 ppb for 30 days could dramatically reduce the latency in the passive avoidance shuttle box assay, indicating memory impairment in zebrafish. The biochemical assays also provided an evidence that the loss of short-term memory in adult zebrafish was due to the dysregulation of the cholinergic system, which may have been caused by the abnormal level of AChE, an enzyme that catalyzes the breakdown of ACh. In summary, we concluded that either the acute exposure of lead at the embryonic stage or the chronic exposure of lead at the adult stage can induce memory impairment in zebrafish.

We also discovered that chronic exposure of PbCl_2_ caused the zebrafish to not only reduce their locomotor activity behavior and decreased aggressiveness in the mirror biting test, but also to exhibit high anxiety-like/freezing behaviors in the 3D locomotion, novel tank exploration, and circadian rhythm locomotor activity tests. Furthermore, abnormal exploratory behavior was also exhibited by the PbCl_2_-treated fish. The assessment of 3D swimming behavior indicated that the exposure with the lowest concentration of PbCl_2_ for seven days made the adult zebrafish less active with a high anxiety index. These findings were concluded from the significant reduction of average speed and average angular velocity. These are also correlated with the significant decrement of total distance traveled by the treated fish. In line with our results, the decrease in locomotor activity and high anxiety were also reported in studies involving mice [31,32]. Another study reported that heavy metal exposure reduced the aggressive behavior of mice [33]. The exact reason why a low dose of PbCl2 displayed a more powerful effect on reducing zebrafish locomotor activity is complex. We proposed that maybe a high dose of PbCl_2_ can trigger the upregulation of metallothionines on buffering the PbCl_2_-induced acute toxicity. On the contrary, a low dose of PbCl_2_ exposure may not yet be enough to trigger metallothionine expressions. More experiments have to be conducted in order to validate this hypothesis in the future studies.

To validate the effect of chronic lead exposure, a variety of animal models have been studied, from a nematode [34], an aquatic model [35], to a rodent model [2]. Lead toxicity is more severe in aquatic animals than in rodents. Lead compounds in recent rodent studies were given by oral gavage that damages the surface of the organs less compared to aquatic animals, of which the lead is dissolved in their environment. We observed the significant increase of the superoxide dismutase (SOD) levels in the brain of PbCl_2_-treated fish. In zebrafish, SOD is required as an antioxidant defense system to reduce accumulated superoxide anions as the consequence of heavy metal overload [36]. The level of malondialdehyde (MDA), a lipid peroxidation product, was also increased significantly. Consistent with the results of previous studies, increased levels of SOD and MDA were observed in the zebrafish embryos treated with chromium (Cr) for 96 h in a concentration-dependent manner (3, 10, and 30 µM) [37]. In addition, the mRNA expression of the gene encoding SOD increased after eight-week exposure to Pb salts [2]. We suggest that the elevation of SOD in the zebrafishes’ brains after chronic PbCl_2_ exposure at a low concentration is a compensatory response of acclimation.

Previous neurotoxicity studies also reported that lead could induce posttraumatic stress disorder in *C. elegans* [34] and children [38,39]. Stress response caused by the heavy metal-induced neurological toxicity was related to elevated levels of catecholamines and cortisol [40,41]. Catecholamines are regulated by the activation of the adrenal medulla. Under stress, an increase in dopamine, norepinephrine, and epinephrine levels occurs [42]. Furthermore, the hormone cortisol has been known to increase as a response to stress [43,44]. In this study, elevated cortisol levels in zebrafish were determined, which explained the high anxiety response in behavioral experiments. Furthermore, we found that the expression of melatonin, the sleeping hormone, showed a significant reduction, which could explain the results of the circadian rhythm locomotor activity [45]. During the dark cycle, the low locomotor activity of PbCl_2_-treated fish with a high freezing time movement ratio was observed. Thus, the PbCl_2_-treated fish could not sleep normally in the dark cycle and exhibited anxiety-like behavior in the day cycle. The dysregulation of the circadian rhythm due to cadmium poisoning has been reported in zebrafish larvae [46]. The circadian rhythm locomotor activity dysregulation caused by lead overload is the first case reported in adult zebrafish. Further studies on evaluating the circadian gene expression at a molecular level are considered necessary to explore its underlying mechanism for circadian rhythm locomotor activity dysregulation.

PbCl_2_ also affected the expression of serotonin and AChE in zebrafish. The low levels of brain serotonin supported the low locomotor activity detected in the PbCl_2_-exposed fish, since serotonin has been reported to play a positive role in regulating locomotor activity in many animals [47,48,49,50,51]. In addition, the reduced expression of serotonin and melatonin was consistent, since both have tryptophan as their precursor. On the other hand, acetylcholine plays an important role on modulating memory and neuromuscular activity. Acetylcholinesterase (AChE) is the enzyme responsible for the breakdown of ACh in the neural synapse. Its activity is usually positively affected by environmental contaminants, such as heavy metals, pesticides, and insecticides [52,53,54]. In this study, we found that AChE activity was compromised after exposure to a low concentration of PbCl_2_ for 30 days. However, cases with enhanced AChE activity were also reported in fish after heavy metal exposure. Romani et al. reported that increased AChE activity was found in the brain and white muscle tissue of bony fish, *Sparus auratus,* after a 20-day exposure to sublethal concentrations of Cu at 0.1 or 0.5 ppm [55]. In another study, the AChE levels in *Seriola dumerilli* were upregulated after Cd exposure at a dose of 50 μg/kg and downregulated at a dose of 100 and 250 μg/kg [56]. In addition, it was discovered that the AChE activity in the brain of the zebrafish increased after chronic zinc exposure at 0.1 ppm for 21 days [57]. We suggest that the inconsistency of the AChE activity response toward heavy metals may be correlated to the nature of the animal model and exposure dose and duration (short-term or long-term). Therefore, memory loss in the PbCl_2_-exposed zebrafish may not be primarily associated with the cholinergic system. More studies on measuring other neurotransmitters that are associated with memory, such as glutamate and glycine, are considered necessary in future studies.

## 4. Conclusions

**Figure 10:**
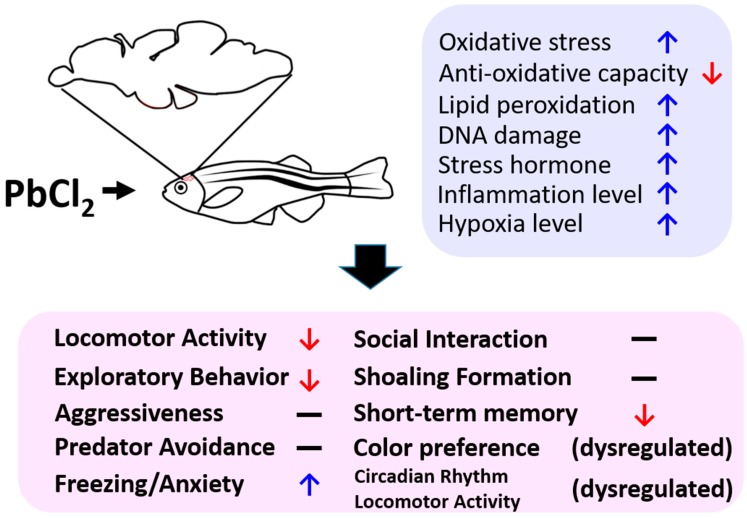
Schematic representation of the biochemical and behavioral signatures detected in chronic and low dose PbCl_2_-exposed zebrafish. The signatures of biochemical (blue) and behavioral (pink) tests are summarized (↑: upregulated, ↓: down regulated).

We performed biochemical and behavioral assessments to evaluate the toxicity of lead in adult zebrafish. The chronic exposure with low concentrations of lead can change the behavior of adult zebrafish. This was indicated by reduced locomotor activity, exploratory response, and short-term memory. In addition, it can elevate anxiety and dysregulate the circadian rhythm and color preference intensity. A biochemical survey demonstrated that the behavioral alterations may be correlated to oxidative stress, lipid peroxidation, and stress (summarized in Figure 10).

## 5. Materials and Methods

### 5.1. Animal Ethics

Wild-type AB strain zebrafish were raised and kept in standard laboratory conditions according to the protocol described by Westerfield [58]. All procedures were approved by The Committee for Animal Experimentation of the Chung Yuan Christian University (Number: CYCU104024, issue date 21 December 2015). All the zebrafish were housed in a recirculating tank system on a 14:10 h light/dark cycle. The water quality was maintained at 28.5 °C (pH 7.2–7.6). For all experiments, we used five-to-six-month-old adult zebrafish.

#### 5.1.1. Lead Chloride Exposure

Lead chloride (PbCl_2_) was purchased from Shanghai Huayi Company (Shanghai, China). A PbCl_2_ stock solution at a concentration of 1000 mg/L (ppm) was prepared in ultrapure water and was stored at 25 °C. Adult fish were exposed to a nominal concentration of 0 (control group), and concentrations of 50 ppb, 100 ppb, and 1000 ppb PbCl_2_ for 24 h, 96 h (acute treatment), 7 days, 10 days, 14 days (sub-chronic treatment), and 30 days (chronic treatment). The water was changed every two days to maintain the lead concentration during incubation.

#### 5.1.2. Adult Neurobehavioral Analysis

Eight behavioral assessments were used to evaluate the neurobehavioral disorders associated with the exposure to low concentrations of lead. The behavioral tests included 3D locomotor activity (for acute and sub-chronic effects), passive avoidance (on day 4, 14, and 30 of PbCl_2_ exposure), aggression (for acute and sub-chronic effects), predator escape/avoidance, social interaction, shoaling, circadian rhythm locomotor activity, and color preference tests (on day 30 of exposure), which are described below.

#### 5.1.3. Zebrafish 3D Locomotor Activity Test

Three-dimensional locomotor activity was tracked at four time points, including 1, 4, 7, and 14 days after the exposure to PbCl_2_, according to a previously described protocol [24]. The 3D locomotion test of six fish was performed using an acrylic tank (20 cm × 20 cm × 20 cm dimension with 15 cm water level).

#### 5.1.4. Novel Tank Test

The novel tank test was conducted to observe the fishes’ ability to acclimate to the novel environment. As described in the previous method, the video recording was started immediately after the tested fish were put into the trapezoid test tank (28 cm × 5 cm × 15 cm) for one minute every 5 min until 30 min passed [59]. Later, six endpoints, which were average speed, freezing time movement ratio, time in top duration, number of entries to the top, latency to enter the top, and total distance traveled in the top, were analyzed.

#### 5.1.5. Aggressiveness Test

Aggressive behavior was determined in adult zebrafish based on the mirror test described in previous studies [59]. The same test tank was filled with ~1.25 L water, where a mirror was placed at the side of the tank. After ~1 min of introduction into the tank, the aggressive behavior, indicated by bites and fast swimming, were recorded for a period of five minutes. Six endpoints, including average speed, mirror biting time percentage, longest duration in the mirror side, freezing time movement ratio, swimming time movement ratio, and rapid movement time ratio, were later calculated.

#### 5.1.6. Predator Avoidance Test

The predator avoidance test is considered as an indicator of predator avoidance behavior in zebrafish. It was carried out based on the previous publication [59]. The same test tank was filled with ~1.25 L water, where a transparent separator was placed in the middle of the tank. The predator convict cichlid (*Amatitlania nigrofasciata*) was placed on one side and the zebrafish were put on the other side. After introduction into the tank, the predator avoidance behaviors, indicated by average speed, predator approaching time percentage, average distance to the separator, freezing, swimming, and rapid time movement ratios, were recorded for a period of five minutes.

#### 5.1.7. Social Interaction Test

A social interaction test was carried out to assess the zebrafishes’ ability to interact with their conspecific. Similar to the predator avoidance test, a glass separator was used in the same size tank as described in the previous publication [59]. After acclimation, the tested fish were introduced in one side of the tank with a conspecific in another side. After video recording, several important endpoints (interaction time percentage, longest duration in the separator side, average speed, and average distance to the separator) were calculated.

#### 5.1.8. Shoaling Test

As an innate behavior, shoaling is one of the social-related behaviors to reduce anxiety and the risk to be captured by predators. Based on the previous publication, a shoaling test was conducted to observe the shoaling formation ability of zebrafish [59]. After acclimation, the behavior of the fish in groups of three were recorded and several endpoints, including average speed, time in top duration, average inter-fish distance, average distance to the center of the tank, average shoal area, and average nearest neighbor distance, were calculated.

#### 5.1.9. Circadian Rhythm Locomotor Activity Test

To evaluate lead-induced disorders in zebrafish locomotor activity during the day and night, we performed a circadian rhythm test based on a previously published procedure [60]. To prevent any external disturbance, the tested fish were moved into a temperature-controlled incubator and kept at 28 °C. A specially designed Light Emitting Diode (LED) lightbox with a conventional and infrared LED array was used as the light source. An infrared-sensitive Charged Coupled Device (CCD) (700–1000 nm detection window) with a maximum resolution of 1920 × 1080 pixels and 30 fps frame rate was used for video recording (3206_1080P module, Shenzhen, China). In this experiment, we recorded the average speed, average angular velocity, and meandering for one minute every hour and used idTracker software [61] to track fish movement trajectories according to our previously published method [24].

#### 5.1.10. Passive Avoidance Test (Short-Term Memory Test)

To assess whether PbCl_2_ could impair short-term memory in adult zebrafish, we carried out a passive avoidance test after 4 days (acute treatment), 14 days (sub-chronic treatment) and 30 days (chronic treatment) of exposure (*n* = 14), referring to previous research with minor modifications [57,62,63]. There were two phases—training and testing—with a 24 h interval between them. In each session, the fish were placed individually in an acrylic tank (20 cm × 20 cm × 20 cm) divided by a door into two compartments of equal size: one black (right side) and one white (left side). Three training sessions were performed and a maximum of two electric shocks were given per session. In each training session, the fish were placed in the white compartment with the door closed for one minute for environment habituation and recognition. Next, the door was lifted, and the fish were allowed to cross over to the dark side of the tank. When the fish swam to the dark area, they immediately received a mild electric shock (5 V, 1 mA) until the fish returned into the white compartment. The fish that completed the training were kept in a small plastic container (15 × 10 cm × 5 cm) with the temperature of water maintained at 28 °C. After 24 h, the fish were placed into the apparatus and tested with the same protocol as the training session, but without the electric shock. The latency to enter the black compartment was observed and its expected increase in the test session was used as an indicator of memory retention.

#### 5.1.11. Color Preference Assay

Color-based experiments are known to be associated with aversion, anxiety, or fear in the zebrafish [64]. The color preference tracking technique is also applied to evaluate neurodegenerative disorders as an index for the preclinical appraisal of drug efficacy and for the behavioral evaluation of toxicity [65]. In this study, we carried out the color preference assay in a 21 cm × 21 cm × 10 cm acrylic tank filled with 1.5 L of filtered water. The tanks consisted of four compartments, and each compartment was divided into two-color combinations among red, green, blue, and yellow. In total, there were six color combinations to determine the color preference of zebrafish. The experiment was done between 10:00 and 16:00. The color preference was recorded with a combination of an IR camera and a HD camera and analyzed using idTracker software [61].

#### 5.1.12. Determination of Neurotransmitters, Neuroendocrine Markers, and Oxidative Stress Parameters

Five randomly selected zebrafish from the control and treatment group were used for biochemical assays. The whole-brain tissue was homogenized on ice in 50 volumes (*v*/*w*) of PBS at pH 7.2 using a bullet blender tissue homogenizer (Next Advance, Inc., Troy, NY, USA). Then, the samples were incubated on ice for 30 min before centrifugation at 12,000× *g* for 10 min. The crude homogenate was stored in 100 μL aliquots at −80 °C for ELISA. The levels of neurotransmitters—namely, serotonin, dopamine, GABA, and Ach—and AChE were measured by using target-specific ELISA kits purchased from a commercial company (ZGB-E1572, ZGB-E1573, ZGB-E1574, ZGB-E1585, ZGB-E1637, Zgenebio Inc., Taipei, Taiwan). The ROS and SOD levels were also determined (ZGB-E1561 and ZG-E1604, Zgenebio Inc., Taipei, Taiwan). The neuroendocrine marker, melatonin, was determined using commercially available ELISA kits (ZG-E1597, Zgenebio Company, Taipei, Taiwan). The target protein content was analyzed according to the manufacturer’s instructions.

#### 5.1.13. Statistical Analysis

All data were presented as mean ± standard error of the mean (SEM). Statistical analyses were performed using the *t*-test, ANOVA, or non-parametric test, depending on data normality for significance determination followed by the least significant difference of multiple comparison tests with The Prism software (Graph pad Software version 7, La Jolla, CA, USA). Statistical significance was considered to be probability (*p*) values less than 0.05.

## Supplementary Materials

Supplementary materials can be found at https://www.mdpi.com/1422-0067/21/5/1844/s1. Video related to 3D locomotion can be found in Supplementary Video S1. Video related to novel tank exploratory behavior can be found in Supplementary Video S2. Video related to aggressiveness in the mirror biting test can be found in Supplementary Video S3. Video related to fear behavior in the predator avoidance test can be found in Supplementary Video S4. Video related to conspecific social interaction can be found in Supplementary Video S5. Video related to shoaling behavior can be found in Supplementary Video S6.
